# Supplementary material for: Besifloxacin Nanocrystal: Towards an Innovative Ophthalmic Preparation
Source: Pharmaceutics. 2022 Oct 18;14(10):2221. doi: 10.3390/pharmaceutics14102221 (PMC9609799; doi:10.3390/pharmaceutics14102221)
Supplement: Supplementary file 1 [file pharmaceutics-14-02221-s001.zip › pharmaceutics-1936726-supplementary.pdf]

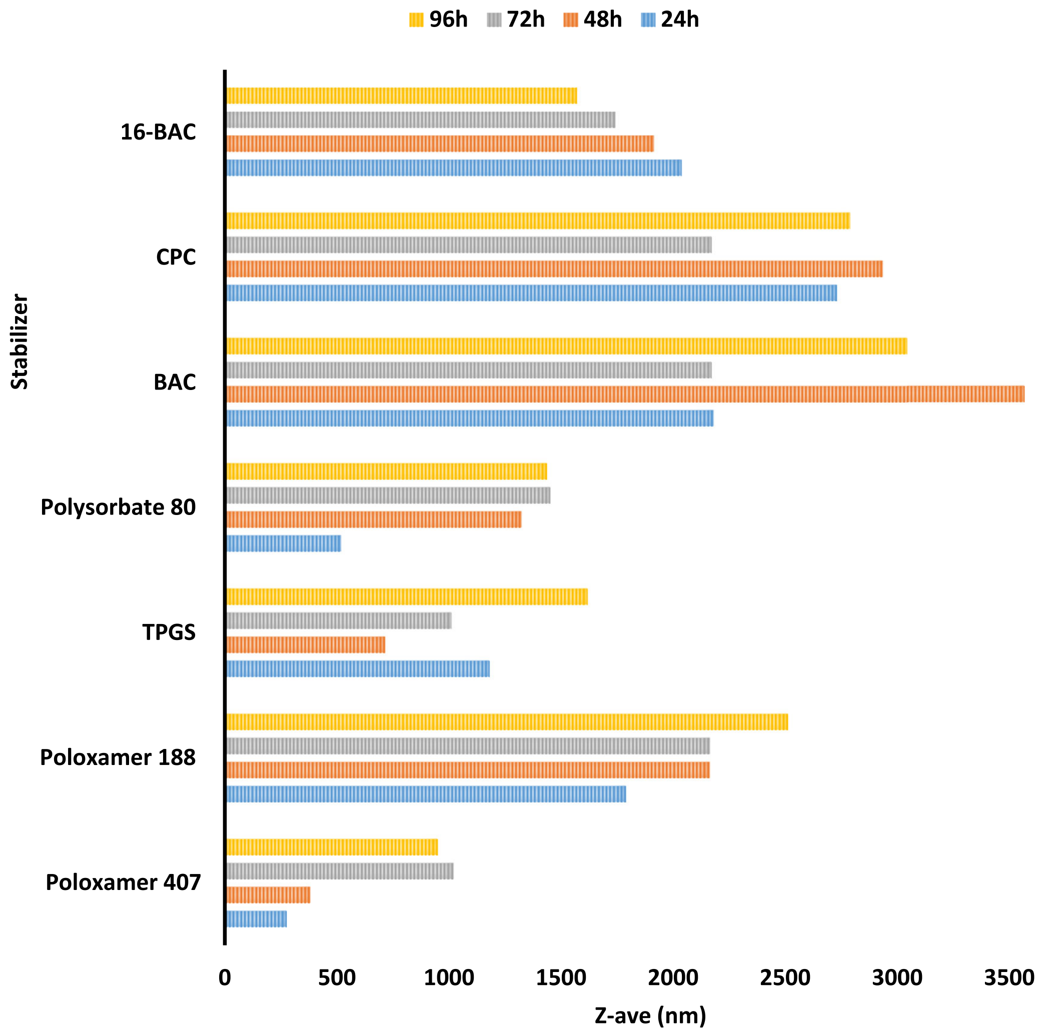


**Figure S1.** Determination of Z-ave (nm) for the first exploratory study. Those stabilizers conventionally used to obtain nanocrystals were tested. The determination of Z-ave was carried out at 24 hours intervals during the milling process (processing time: 96 hours). Conditions: 1200 RPM, 30% (wt%) zirconium beads. Concentration: stabilizer and besifloxacin 0.6 wt% (1:1). BAC: benzalkonium chloride; CPC: cetylpyridinium chloride; TPGS: tocopherol polyethylene glycol succinate; 16-BAC benzyldimethylhexadecylammonium chloride.

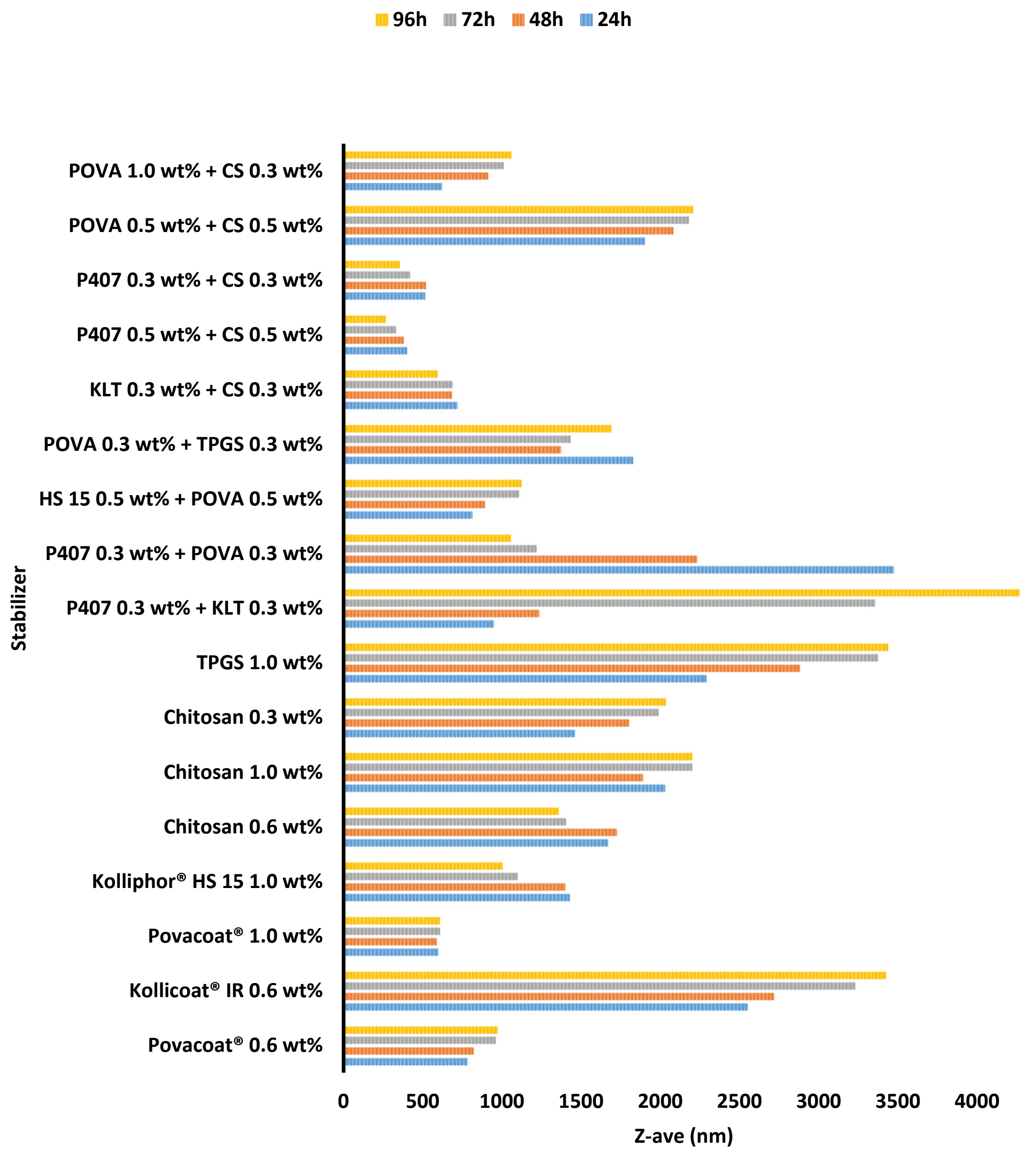


**Figure S2.** Determination of Z-ave (nm) for the second exploratory study. New and promising stabilizers, different combinations. The determination of Z-ave was carried out at 24 hours intervals during the milling process (processing time: 96 hours). Conditions: 1200 RPM and 30% (wt %) zirconium beads. Besifloxacin 0.6 wt %. TPGS: tocopherol polyethylene glycol succinate; P407: Poloxamer 407; KLT: Kollicoat® IR; POVA: Povacoat®; HS 15: Poloxamer HS 15; CS: chitosan.

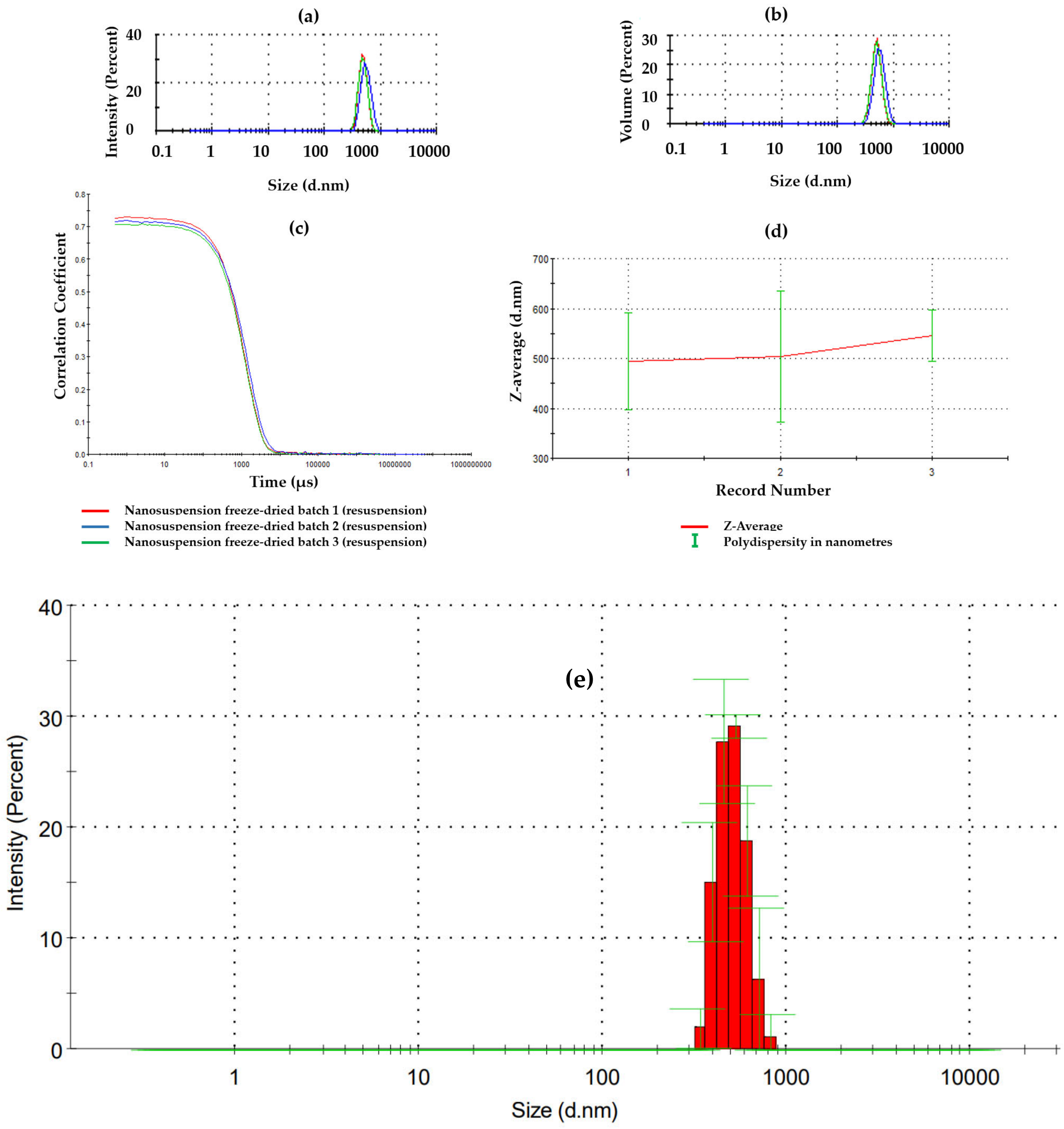


**Figure S3:** Freeze-dried besifloxacin nanosuspension DLS measurements: (a) Size distribution by intensity; (b) Size distribution by volume; (c) Correlogram; (d) Z-average trend; (e) Statistics graph *n* =3.

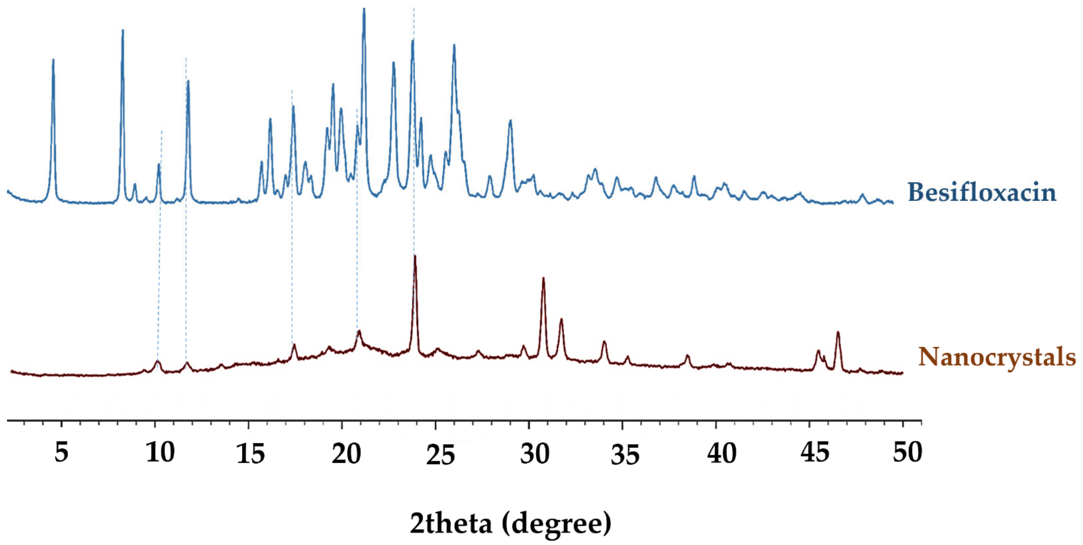


**Figure S4:** X-ray diffraction of besifloxacin (raw material) and nanocrystals (freeze-dried). $n = 3$.

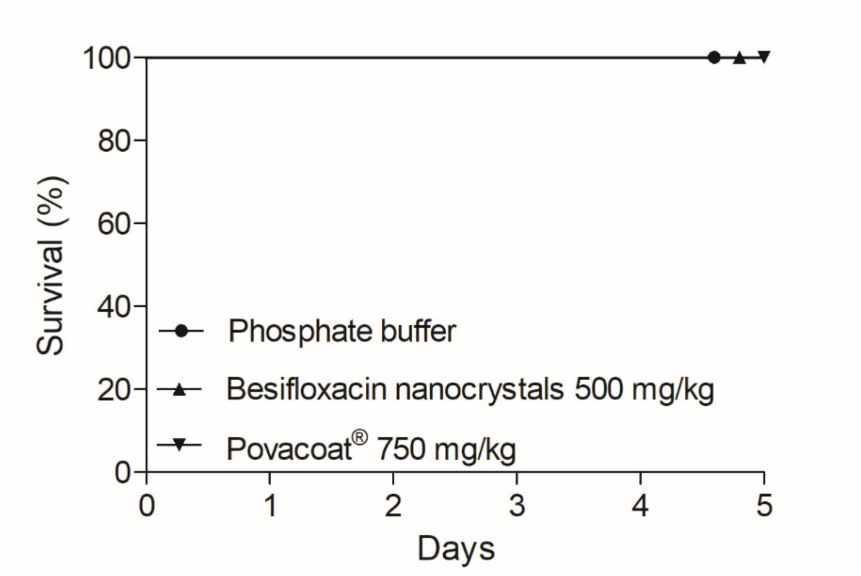


**Figure S5.** Toxicity of besifloxacin nanocrystals (500 mg/kg) and Povacoat® (750 mg/kg) in a larval model of *Galleria mellonella*.

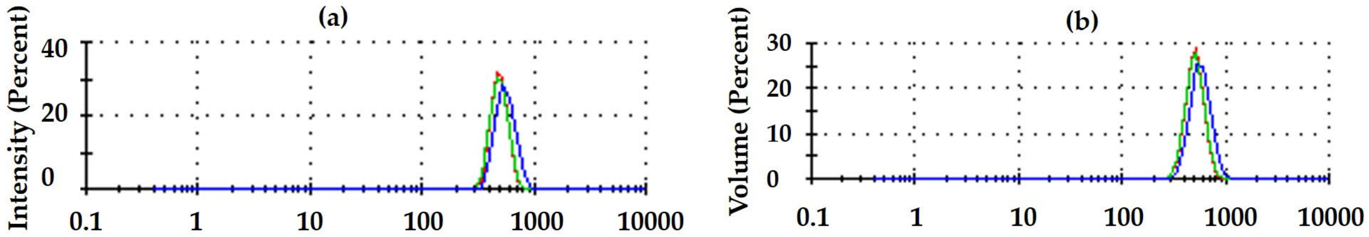


**Figure S6:** Particle distribution (monomodal), accelerated stability: 90 days. Study condition: 40°C ± 2°C / 75% RH ± 5% RH. (a) Size distribution by intensity; (b) Size distribution by volume. $n = 3$.

**Table S1**. Stability of the average hydrodynamic diameter (Z-ave) in nm, in the time intervals: immediately after milling (T0), 7 days, 15 days and 30 days. Process time 4 days. Storage condition: 25°C.

| | Storage time (25°C) | | | |
|---|---|---|---|---|
| Stabilizer | T0 | 7 days | 15 days | 30 days |
| Povacoat® 1.0 wt% | 607.2±11.56 | 662.2±10.7 | 660.9±15.5 | 679.4±1.8 |
| Poloxamer P 407 0.6 wt% | 337.7±14.6 | 418.5±9.9 | 415.5±7.9 | 450.7±10.3 |
| KLT 0.3 wt% + CS 0.3 wt% | 594.7±22.57 | 1205.0±173.9 | - | - |
| Polysorbate 80 0.6 wt% | 518.9±9.3 | 1100.0±33.5 | - | - |
| TPGS | 796.5±56.7 | 1570.0±191.3 | - | - |
| P407 0.5 wt% + CS 0.5 wt% | 267.6±1.5 | 398.0±5.6 | 428.8±10.8 | - |
| P407 0.3 wt% + CS 0.3 wt% | 355.3±7.1 | 570.1±18.67 | 479.2±19.35 | - |

(-): unmeasured; TPGS: tocopherol polyethylene glycol succinate; P407: Poloxamer 407; KLT: Kollicoat® IR; CS: chitosan.

**Table S2:** Nano-based besifloxacin delivery: type, composition, properties, antimicrobial activity and, physical-chemical stability condition.

| Type | Components | Besifloxacin concentration | Size | PdI | Zeta potential (mV) | Antimicrobial Activity | Physical-chemical stability | Ref. |
|---|---|---|---|---|---|---|---|---|
| LP | LP PC<br>LP PC: SPM | 335.81 - 467.84 μg/mL | 175.4 - 177.2 nm | 0.019 ± 0.011<br>0.075 ± 0.021 | +19.5 and<br>- 5.7 | *S. epidermidis:*<br>MIC: 0.156 μg/mL<br>*P. aeruginosa:*<br>MIC: 0625 μg/mL | 6 °C for 30 days | [17] |
| NE | Triacetin (oil), Cremophor® RH 40, and Transcutol®P | 0.2 wt% | 14.0 nm | 0.145 - 0.217 | - | comparative efficacy of lower drug-loaded NE (0.2%) versus 0.6% besifloxacin suspension against<br>*P. aeruginosa* and *S. aureus* | heating–cooling<br>(2–8 °C) and 40 °C<br>for 48 h + 25 °C,<br>at least 48 h,<br>13,000 rpm for 30 min | [18] |
| NLC | Sulfobutyl ether beta-cyclodextrin and chitosan | 0.005 % (m/m) | 13.6 – 16.1 nm | 0.107 - 0.181 | +33.8 ± 1.9 | *P. aeruginosa:*<br>MIC: 0.031 μg/mL | 4 °C for 30 days | [19] |
| NF | HP-β-CD; PCL/PEG; sodium alginate or thiolated sodium alginate | 40 μg / cm² fiber insert | thickness: 0.66 - 0.83 mm; diameter:<br>6.6-6.8 mm | - | - | bacterial keratitis in rabbit eyes was reduced upon single-dose application compared to multiple dosing with the commercial drug.<br>Against *P. aeruginosa* | - | [20] |

LP: Liposome; LP PC: Phosphatidylcholine; LP PC: SPM: Phosphatidylcholine and spermine; HP-β-CD: Hydroxypropyl-beta-cyclodextrin; PCL/PEG: Poly(caprolactone)/polyethyleneglycol MIC: Minimal inhibitory concentration; NE: Nanoemulsion; NF: Nanofibrous; NLC: Nanostructured lipid carrier; PdI: polydispersivity index, (-) not disclosed.
